# Evaluating the Impact of a Virtual Skin Cancer Awareness Workshop for 16-19-Year-Olds

**DOI:** 10.7759/cureus.74664

**Published:** 2024-11-28

**Authors:** Maya Dyson, Anna Corriero

**Affiliations:** 1 Internal Medicine, Sandwell and West Birmingham Hospitals NHS Trust, Birmingham, GBR; 2 Medical Education, NHS Lothian, Edinburgh, GBR

**Keywords:** dermatology, medical education, melanoma, skin cancer, widening participation

## Abstract

Introduction

The incidence of malignant melanoma (MM) in the United Kingdom (UK) has significantly increased in recent years and is expected to continue to rise over the next decade. Despite the preventable nature of most MM cases, existing evidence suggests that public health education around skin cancer and sun safety is often suboptimal, particularly for secondary school populations. Unlike primary school curricula, there is no national guidance to mandate the teaching of this topic in secondary school. Early intervention through education can encourage sun-safe practices, and therefore potentially reduce the risk of MM developing. The primary aim of this study was to improve awareness of skin cancer, including its risk factors and preventive measures, amongst sixth-form students.

Methods

An online workshop was organised by In2MedSchool, a national widening access charitable scheme for aspiring medical students, in collaboration with the Karen Clifford Skin Cancer Charity. Plan-Do-Study-Act methodology was utilised to design and evaluate a skin cancer awareness and sun safety workshop. It addressed key topics such as MM risk factors, preventive measures, and the widely used 'ABCDE’ assessment of MM. A total of 73 students from across the UK attended the virtual workshop. To maximise engagement, visual aids, low-stakes quizzing, and an interactive chat function were utilised.

Participants completed anonymous pre- and post-intervention questionnaires, assessing their confidence and knowledge in understanding skin cancer risk factors and sun-safe behaviours. Confidence levels were measured using a seven-point Likert scale, and knowledge was evaluated through a ten-question assessment. Statistical analyses, including averages, medians, interquartile ranges (IQR), and Wilcoxon signed-rank tests were performed to assess the changes in participants’ knowledge and confidence.

Results

Before the intervention, participants reported an average confidence score of 4.07 in understanding skin cancer risk factors, which significantly improved to 6.39 after the workshop, representing a 57.09% increase (p < 0.00001). Confidence in applying protective measures increased by 45.67%, from 4.51 to 6.57 (p < 0.00001). In addition, most students expressed feeling underprepared by existing sun safety programs at both primary and secondary school levels.

Knowledge assessments demonstrated significant improvement, with average scores increasing from 7.49 pre-intervention to 9.10 post-intervention (21.46% improvement, p < 0.00001). The median score increased from 8 (IQR: 3) to 9 (IQR: 1) following the workshop, demonstrating a notable increase in understanding of skin cancer risk factors, protective behaviours, and early detection methods.

Conclusion

Our findings indicate that many secondary school students feel unprepared regarding existing skin cancer awareness and sun safety programs in school. This study provides evidence to suggest that targeted, interactive workshops have the potential to improve awareness and knowledge of this important topic in the short term. However, participants in this cohort are likely to have an established interest in medicine and healthcare, beyond that of their wider peer group. This limits the broader application of our findings. Further research that explores the longer-term impact of such interventions, including changes in attitudes and behaviours, is recommended.

## Introduction

The incidence of malignant melanoma (MM) in the United Kingdom (UK) has risen significantly in recent years, with rates projected to continue increasing over the next decade [1,2]. This is widely considered to be the result of a number of factors, including increased exposure to ultraviolet radiation (UVR) over time, which is one of the key risk factors in the pathogenesis of all three major forms of skin cancer [3,4]. The burden of MM is considerable; in the UK it is the fifth most common cancer in women and fourth most common in men. Recent data from Cancer Research UK has revealed that MM is one of the most commonly diagnosed cancers in young people [5]. Existing evidence suggests that skin cancer awareness is suboptimal among adolescents, with the continued use of sunbeds among teenagers and young adults posing a growing public health concern [6,7]. The majority of cases are thought to be preventable and public health education is pivotal [2,8]. Damage from UVR is cumulative and the risk of developing MM increases with age [3]. Whilst individuals may experience sunburn during their childhood or teenage years, they may only face the long-term consequences of this as adults. As a result, educating younger populations is an integral part of effective primary prevention [9]. Such interventions have shown the potential to help establish sun-safe practices early in life [10-12]. In the UK, the topics of skin cancer awareness and sun safety are included in the national curriculum for primary schools, as set by the Department for Education [13]. This mandates that state-run primary schools provide education on these matters. However, independent and faith primary schools are exempt from this and have more autonomy over their curricula. Notably, this topic is lacking from equivalent national secondary school curricula. Consequently, there is likely significant variation in how these topics are taught in schools across the country. On review of the literature, a lack of tailored interventions specifically for secondary school-age children appears to be a widespread issue [12,14]. The primary aim of this project was to improve awareness of skin cancer, including its risk factors, and promote sun-safe behaviours amongst sixth-form students. The secondary aim was to offer students from widening participation backgrounds, with aspirations to study medicine, a valuable learning experience that could support their interest in healthcare and science.

## Materials and methods

An online workshop was planned and conducted by junior doctor volunteers from In2MedSchool in collaboration with the Karen Clifford Skin Cancer Charity. Plan-Do-Study-Act (PDSA) methodology was utilised to design, implement, and evaluate the skin cancer awareness workshop. This four-step process provided a systematic framework to address the needs and learning outcomes of participants. Its cyclical nature enables continuous refinement of quality improvement-focused interventions. A full PDSA cycle was completed over a six-week period.

The workshop involved a 90-minute interactive lecture, which utilised visual aids (including slides that incorporated photographs, graphs and diagrams), low-stakes quizzing and an active chat function. The lecture was divided into five sections based on the following topics: an overview of skin cancer epidemiology, its risk factors, the different types of skin cancer, the "ABCDE criteria" for assessing MM, and sun-safe practices [15]. Each section concluded with a short multiple choice quiz, which participants responded to anonymously, to check understanding and reinforce new knowledge. Following this, participants had the opportunity to ask questions on each topic, via the chat function. 

Eligibility for participation was restricted to students enrolled in the In2MedSchool charity widening access scheme, which supports students from underrepresented backgrounds with the process of applying to medical school through mentorship and educational events. Inclusion criteria required participants to be 16-19 years old, UK residents, enrolled in the In2MedSchool widening access scheme, and possessing sufficient English proficiency to engage with the workshop. Exclusion criteria were as follows: individuals outside the aforementioned age range, residing outside the UK, not meeting widening participation criteria, or lacking reliable internet access. Participants completed a pre- and post-intervention questionnaire (see Appendices), with perception and confidence-based questions assessed using a seven-point Likert scale. This included perception of previous skin cancer and sun safety teaching in both primary and secondary school. In addition, a 10-question assessment was used to quantify changes in knowledge of key domains such as skin cancer risk factors, skin protective measures and characteristic features of MM. Statistical analysis included averages, medians, interquartile ranges (IQR), and the Wilcoxon Signed-Rank Test. The latter was chosen for statistical analysis as it is a non-parametric test and enabled comparison of paired data collected from pre- and post-intervention questionnaire responses. 

No ethical approval was required as data was collected anonymously as part of a service evaluation. On signing up to participate, students were informed that anonymous data would be collected during the study and that it may be stored and used for future research purposes. They were provided with the option to opt out of this process if they did not wish for their data to be included.

## Results

A total of 73 students participated in the live workshop and all completed the pre- and post-intervention questionnaires. Results of the pre-intervention questionnaire highlighted that the majority of students felt inadequately prepared by their primary and secondary school education regarding skin cancer awareness and sun safety (Table 1).

**Table 1:** Participant perception of previous skin cancer and sun safety teaching 1 = strongly disagree, 2 = disagree, 3 = somewhat disagree, 4 = neither agree nor disagree, 5 = somewhat agree, 6 = agree 7 = strongly agree

Questionnaire statement	Average Likert score	Median Likert score	IQR
I felt prepared by teaching during primary school regarding sun safety and/or skin cancer	2.75	3	3
I felt prepared by teaching during secondary school regarding sun safety and/or skin cancer	3.34	3	3

Comparison between the pre- and post-intervention questionnaire responses demonstrated a significant increase in all assessed domains related to perceived confidence (Table 2). The mean score of the knowledge-based assessment increased from 7.49 to 9.10 (Table 3). Both the increase in self-reported confidence and the improvement in knowledge test scores post intervention were statistically significant. 

**Table 2:** Self-reported confidence in skin cancer prevention-related domains 1 = strongly disagree, 2 = disagree, 3 = somewhat disagree, 4 = neither agree nor disagree, 5 = somewhat agree, 6 = agree 7 = strongly agree

Questionnaire statement	Likert score		% change +/-	P value
	Pre-intervention	Post-intervention		
I feel confident in understanding the risk factors for developing skin cancer	4.07	6.39	+57.09	p<0.00001
I feel confident in my knowledge of the 'ABCDE' concept relating to skin cancer	2	6.33	+216.39	p<0.00001
I feel confident I know what to do to protect my skin from skin cancer	4.51	6.57	+45.67	p<0.00001

**Table 3:** Mean score of knowledge-based assessment

Raw mark (maximum 10)		% change +/-	P value
Pre intervention	Post intervention		
7.49	9.10	+20	p<0.0001

Participant feedback on the workshop was overwhelmingly positive with 83% responding with “strongly agree” when asked to what extent they agreed that “This session was valuable for my learning” on the post-intervention questionnaire. The average response to this question was 6.75, the median was 7, and no students responded with a score lower than 5. 

## Discussion

Perceptions of preparedness in skin cancer awareness 

The results highlight the prevalent perception among participants that they are underprepared regarding skin cancer awareness and sun-safe practices through both their primary and secondary education. Concerningly, 54.75% of participants responded with “strongly disagree”, “disagree” or “somewhat disagree” when asked to what extent they agreed that “I feel prepared by teaching during primary school regarding sun safety and skin cancer”. Similarly, 42.47% of participants responded in the same manner when asked to what extent they agreed that “I feel prepared by teaching during secondary school regarding sun safety and skin cancer”. Interestingly, within this cohort, the perception of secondary school teaching on this topic was superior to that of primary school teaching. This may be considered somewhat surprising given that sun safety and skin cancer awareness are explicitly included in the national curriculum for primary schools, but not that of secondary schools [13]. This suggests that some secondary schools might be incorporating sun safety in their curriculum, despite it not being mandated at the government level. This discrepancy in perceived preparedness could be partially attributed to the recency effect, where participants recall more recent information more vividly, leading to a perception of being better prepared by secondary school education [16]. Additionally, this may suggest potential gaps in the retention and long-term impact of primary school teaching on sun safety and skin cancer awareness. Perception is highly subjective; however, our findings prompt the need for further exploration of the underlying reason for such widespread dissatisfaction and whether this reflects a scarcity of such teaching or the perceived ineffectiveness of existing programs. 

Knowledge as a predictor of behavioural change 

Comparison between pre- and post-intervention questionnaire responses shows a significant improvement in both knowledge and confidence among participants after attending the workshop. Confidence and perception-based questions are subjective and, unqualified, may not be a reliable indicator of the effectiveness of such an intervention [17]. However, the profound increase in assessment scores suggests that the intervention was effective in improving knowledge about skin cancer and sun-safe practices, in the short term. This supports findings from several systematic reviews examining the impact of similar interventions, which highlight that targeted educational initiatives often result in significant short-term increases in knowledge of this topic amongst participants [10,12,18]. It is likely that these findings are influenced by the previously discussed recency effect [16]. It is noted that there is a paucity of studies which include longitudinal assessment in their design, and this highlights an area of recommended future research [11]. Studies which involve longitudinal assessment are likely to require more complex design and ethical approval, which may make them more challenging to implement. Perhaps a more achievable alternative that future researchers may wish to consider is to incorporate assessment after a week or month. Although this is unlikely to provide the same insights as longer-term assessments, for example after six months or a year, it would likely provide further indication of the educational impact beyond the immediate post-intervention phase. Interestingly, Calco highlighted that of those studies which included longer-term assessment in their design, most demonstrated a sustained improvement in knowledge, and some attitudes and behaviours [12]. The same systematic review identified that most studies did not include an assessment of perceived confidence in their design, despite a strong correlation between increased participant confidence and positive behavioural change. Assessing confidence alongside other, more robust, outcome measures has the potential to be a useful indicator of the success of an intervention. 

It is difficult to predict whether the significant increase in knowledge seen amongst participants had any meaningful impact on attitudes and behaviours, as this was not included in the study design and is one of its more significant limitations. Ultimately, practising sun-safe behaviours is what has the potential to improve health outcomes [1,2]. Commonly, knowledge is the only assessed outcome of such interventions [10]. This is possibly a result of behaviours and attitudes being somewhat more complex to assess and measure. A systematic review from 2021 noted that targeted educational interventions frequently result in an increase in knowledge of skin cancer and sun safety; however, improvement in adopting preventative behaviours is often negligible [18]. However, two more recent systematic reviews have found convincing evidence that such interventions can have a significant positive impact, beyond knowledge, amongst secondary school-age students [10,12]. Furthermore, a number of large studies have reported that a more significant increase in knowledge is strongly associated with both stronger reported intention to follow sun-safe practices and actual behavioural change [19,20]. Therefore, it could be argued that knowledge remains a useful measure. Further scoping reviews which consider the design of the intervention, with a particular focus on educational and psychological theory, may be helpful in identifying what strategies are more conducive to attitudinal and behavioural change. 

Educational theory and intervention design 

Interestingly, a large, recent systematic review has highlighted that evidence-based educational practice is not routinely incorporated into skin cancer prevention programs, which further prompts more rigorous design of such interventions [12]. Overall, it is widely recommended that interventions are included as part of a longitudinal program throughout school [12,19,21]. It is suggested that repeated, skin cancer awareness and sun safety interventions are required for significant behavioural change amongst participants [18]. This is in keeping with the educational theory of spaced practice, which suggests that staggering educational interventions can improve long-term retention of learned knowledge [22,23]. Due to the logistical limitations, this was not possible to facilitate in this instance. However, participants were provided with online access to all resources used during the session and encouraged to revisit. A follow-up email was sent to participants one month after the intervention as a further reminder. Future studies may wish to consider both longer-term interventions and longitudinal assessment to assess whether the impact of such teaching is sustained. 

Longitudinal programs may also be conducive to reducing the cognitive load of individual teaching sessions, a concept which considers the limitations of the working memory in the context of the learning process [24]. This is crucial for participant engagement, and for enhancing retention of new information. Other elements of the intervention design aimed to address the cognitive load of participants. The manner in which the workshop was structured, with distinct sections for each topic, and the opportunity for a number of short breaks was intentional. Short breaks during a teaching session, or 'micro-breaks' as they are sometimes referred to, have been shown to help improve the overall performance of learners [25]. Evidence suggests that educational interventions which incorporate short breaks can help improve attention and concentration as a result of reducing cognitive load and the risk of fatigue [26]. This may also improve overall satisfaction amongst participants, which could improve engagement with future initiatives.

It is suggested that active learning strategies in this context are particularly effective for improving sun-safe behaviours and attitudes, in addition to knowledge [12,18]. This reflects wider thinking that teaching is most effective when it is a dynamic, learner-centred process and this was considered in the design of the workshop. This event is live and interactive, with regular opportunities for low-stakes quizzing, in the form of regular polls throughout the session that signified the end of a particular topic. There is also an active chat function used throughout, to promote engagement and improve the retention of knowledge [26]. These activities can help promote higher cognitive processes, such as analysis and evaluation of information, which are more conducive to long-term retention of knowledge [27]. The format allowed students to respond to questions and interact anonymously, without concerns of publicly providing an incorrect answer. This helps those delivering the session establish rapport with learners, and also provides a safe learning environment that encouraged reflection and asking of questions. 

The role of participant demographics and engagement 

Participants were all involved in the In2MedSchool mentorship and educational program, which is designed to support school students aged 16-19 with established widening participation status who have aspirations to study medicine. Therefore, participants may have been more likely to have an underlying interest and, potentially, knowledge of the subject matter. This could also have positively impacted their engagement and motivation, and therefore our results may not be representative of the wider population of this age range. Due to the single-cohort design of the intervention, it is not possible to determine the extent to which this potential bias may have influenced the results. This limitation has crucial implications for the generalisability of our findings. Future studies incorporating control groups or cohorts with varied levels of interest in medicine and healthcare would help to better understand the influence of this factor and broaden the applicability of such interventions. This could provide insights into whether our findings hold true in the broader population. Interestingly, some studies have found that educational background is an independent predictor of baseline skin cancer awareness knowledge [28].

It is suggested that tailoring interventions according to participant demographics is more likely to result in behavioural and attitudinal change [12]. Participants' presumed established interest in healthcare and medicine was considered in the design and delivery of this workshop. The workshop covered the psychosocial impact that skin cancer can have on patients [29]. This included discussion of the potential distress caused by visible skin damage and scarring as a direct result of skin cancer itself or associated surgical interventions, thus offering a more holistic understanding of this group of conditions. This approach aimed to contextualise the issues of individual patients within the larger public health framework, in order to further emphasise the important role that education and early intervention play in reducing the impact of skin cancer.

On review of the wider literature, it has been seen that some authors have proposed that educational programs which have highlighted the association between UVR and photoaging, in addition to the potential cosmetic impact of skin cancer, may be particularly effective amongst adolescents [6,12]. It is noted that social and cultural influences on the perception of tanned skin are commonly attributed to the persistent use of sunbeds and sun-seeking behaviour amongst many adolescents and young adults [10,12]. Concerningly, this behaviour has been shown to persist even when there is reasonable awareness of the associated risk of skin cancer [21,30]. This suggests that an increase in knowledge alone may be insufficient to encourage the adoption of sun-safe behaviours [7]. Future research that addresses the gap in understanding whether short-term knowledge improvements can translate into behavioural change would provide valuable insights. The topic of sun-tanned skin was mentioned in parts of the workshop covering skin cancer risk factors and sun safe practices. This topic was also included in two questions in the knowledge assessment. However, the suggested pervasive widespread preference for tanned skin amongst adolescents may warrant a more direct, targeted approach, beyond the scope of this intervention, given the complexity of the issue and wider socio-cultural context [7,30]. In summary, when designing an educational intervention with a similar aim, consideration of the age, educational stage and other psychosocial factors which may affect the engagement and motivation of participants is encouraged. 

## Conclusions

The majority of participants reported feeling inadequately prepared in terms of skin cancer awareness and prevention by their formal education, suggesting possible gaps and inconsistencies in how this topic is currently addressed in school. This prompts the need for a thorough evaluation of the underlying reasons for this and a review of current educational approaches, including the design and delivery of skin cancer awareness programs. The statistically significant improvement in both knowledge and confidence following this targeted educational intervention demonstrates its effectiveness in improving understanding of a number of key topics including risk factors, sun-safe behaviours, and the identification of MM. Initiatives like the one evaluated in this study have the potential to effectively supplement existing local curricula. As described, such initiatives should ideally be delivered in addition to, or as part of, a more comprehensive and longitudinal program in order to maximise impact in terms of knowledge retention and attitudinal and behavioural change. Moreover, in order to fully evaluate the effects of such interventions longitudinal assessment is essential, as is specific evaluation of attitudes and behaviours. These methods would provide valuable insights into the most effective, evidence-based educational strategies for promoting prevention and early detection of skin cancer. However, it is acknowledged that incorporating such components into the design of an intervention would significantly add to the logistical complexity.

Our findings should be interpreted in the context of a number of limitations; notably, the pre-existing interest in medicine amongst participants and the possible influence on motivation of individuals and engagement with the teaching material. This may have positively affected both baseline knowledge and the overall impact of the session including the acquisition of knowledge. Specific regard for participant demographics, and tailoring interventions accordingly, is widely recommended. As discussed, the background and motivating factors of participants involved in this study were considered in its design and delivery, and this may have contributed to its impact. Future research should explore how similar interventions can be adapted for broader, more diverse, groups including those without a pre-existing interest in medicine and low baseline knowledge of the subject matter. Our results suggest that further research efforts are required to enhance skin cancer education for secondary school children. This study provides promising evidence of the short-term effectiveness of targeted educational interventions on both confidence and knowledge related to skin cancer and sun safety. However, participant demographics and the absence of longitudinal assessment limit the wider application of our findings.

## Appendices

Questionnaire

The following questionnaire was completed by participants both before and and after the intervention. *1) Please Rate the Below Statements from 1 - 7, Using the Following Scale:* 1 = strongly disagree, 2 = disagree, 3 = somewhat disagree, 4 = neither agree nor disagree, 5 = somewhat agree, 6 = agree 7 = strongly agree a) I feel confident in understanding the risk factors for developing skin cancer b) I feel confident in my knowledge of the 'ABCDE' concept relating to skin cancer c) I feel confident I know what to do to protect my skin from skin cancer d) I felt prepared by teaching during primary school regarding sun safety and/or skin cancer e) I felt prepared by teaching during secondary school regarding sun safety and/or skin cancer

2) Please Respond With True/False to the Following Statements:

a) A sun tan helps protect your skin from damage caused by ultraviolet radiation (UVR) b) Melanoma (often the most serious form of skin cancer) does not develop from pre-existing moles/ birthmarks (i.e. it is always something new on the skin) c) You can only get a sunburn when it is hot outside d) A single episode of sunburn increases the risk of developing skin cancer (including melanoma) e) Waterproof sunscreen provides adequate protection to last a full day f) Darker skin tones do not develop melanoma (often the most serious form of skin cancer) g) The majority of skin cancer deaths are preventable h) Sunscreen is most effective when applied immediately before going outdoors i) The DNA damage from UV rays can be reversible j) Using a sunbed to get a tan is safer than normal sunbathing
